# Perceptions of final-year medical students and interns toward pursuing a career in critical care medicine

**DOI:** 10.3389/fmed.2024.1502997

**Published:** 2025-01-10

**Authors:** Mohammed Ageel

**Affiliations:** College of Medicine, Jazan University, Jazan, Saudi Arabia

**Keywords:** critical care medicine, medical students, specialty choice, perceptions, work-life balance, career counseling, Jazan University, medical education

## Abstract

**Background:**

Critical care medicine (CCM) faces challenges in attracting new physicians due to its demanding nature. Understanding medical students’ and interns’ perceptions of CCM is essential to address physician shortages and improve medical training.

**Objective:**

To evaluate the factors influencing specialty selection and explore perceptions of final-year medical students and interns toward CCM at Jazan University.

**Methods:**

A cross-sectional survey using convenience sampling was conducted. Participants completed questionnaire assessing factors influencing career choice and perceptions of CCM. Chi-squared tests analyzed the associations between demographic variables and perceptions.

**Results:**

The study included 165 medical students (80% interns, 20% final-year students), primarily male (56.36%) and single (90.91%), with a mean age of 24.56 ± 1.46 years. The most influential career factors were job security (11.65%), enthusiasm/commitment (10.36%), and acceptable working hours (10.36%). Most students (81.82%) found CCM intellectually challenging, while 76.36% recognized its financial benefits. However, only 29.09% were considering a career in CCM, with concerns about work-life balance (34.54%) and family time (32.73%). Interns were more likely than final-year students to view limited working hours positively (*p* = 0.046), perceive greater colleague prestige (*p* = 0.035), expect private practice opportunities (*p* = 0.004), anticipate higher salaries (*p* < 0.001), and acknowledge the shortage of staff in CCM (*p* = 0.009).

**Conclusion:**

There is a tension between the intellectual and financial benefits of CCM and its lifestyle demands. While students recognize its rewards, concerns about work-life balance and staffing challenges discourage many from pursuing the specialty. Tailored career counseling, mentorship, and addressing lifestyle concerns in medical curricula may improve interest in CCM.

## 1 Introduction

Critical care medicine (CCM) is a vital medical specialty focused on the management of patients with life-threatening conditions. Physicians in this field require extensive medical knowledge, advanced procedural skills, and the ability to make rapid, high-stakes decisions in complex, acute care settings (1). Despite its critical importance, there is a growing shortage of CCM specialists, posing a significant threat to healthcare systems worldwide. This shortage has the potential to compromise patient care, especially in intensive care units (ICUs), where timely, expert management is essential (2). Understanding how both final-year medical students and interns perceive CCM is crucial to fostering interest in the field and addressing this workforce gap.

For both final-year medical students and interns, CCM presents both significant learning opportunities and formidable challenges. The ICU is a high-pressure environment where patients often require advanced interventions and continuous monitoring (3). Final-year students, during their clinical rotations, are exposed to critically ill patients, which provides them with an opportunity to participate in complex procedures and decision-making processes. However, this experience can also be overwhelming due to the severity of illness, the rapid pace of care, and the emotional toll of managing high mortality rates (4, 5). Similarly, interns, who often have increased clinical responsibilities, face additional pressures, such as balancing patient care with skill acquisition in a stressful environment. For both groups, this exposure may either inspire a career in CCM or dissuade them due to the intense demands (6).

Previous research has explored medical trainees’ experiences in CCM. One systematic review found that students appreciate the opportunity to work closely with ICU teams, manage critically ill patients, and gain procedural experience (4). However, these same students often report feeling emotionally drained, face ethical dilemmas, and struggle with feelings of inadequacy in the ICU environment. These challenges may differ between final-year students and interns, as interns may experience more direct responsibility for patient care. Additionally, studies focused on interns have found that, despite initial feelings of unpreparedness and stress, the ICU experience can increase their interest in pursuing a career in CCM (7).

The internship period, in particular, is pivotal in shaping a young doctor’s career trajectory. It is during this time that interns consolidate their clinical skills, solidify their professional identity, and explore potential specialties (8). Similarly, final-year students, while earlier in their training, may begin to form strong impressions of specialty areas during their clinical rotations. Both groups’ experiences in CCM could influence whether they consider it as a career choice. This is especially relevant given the increasing global demand for CCM physicians (2, 5). Understanding the perspectives of final-year students and interns can provide insights into the factors that attract or deter them from pursuing CCM, which may help guide educational reforms aimed at mitigating the current physician shortage.

Insights from these groups are also essential for optimizing ICU rotations and educational content during these formative training periods (7, 8). Their feedback can inform curriculum development and mentorship programs, allowing medical schools to target areas where trainees feel underprepared and reinforce areas of confidence. Such targeted interventions can improve both the technical and emotional preparedness of trainees for CCM, ensuring a better fit between their career aspirations and the demands of the specialty (9–11).

However, limited data exists on the specific factors influencing final-year students’ and interns’ perception toward pursuing a career in CCM. Therefore, this study seeks to evaluate the factors influencing specialty selection and explore perceptions of final-year medical students and interns toward CCM at Jazan University. This study provides insights into the perceptions of final-year medical students and interns regarding CCM as a career specialty, focusing on factors influencing their career decisions within the context of Saudi Arabia. Unlike previous studies, which primarily examined medical trainees’ general preferences for medical specialties, this research highlights the unique challenges and opportunities associated with CCM, including its intellectual demands, financial benefits, and work-life balance concerns. By identifying specific barriers, such as concerns about lifestyle compatibility and the emotional toll of CCM, the study offers targeted recommendations for medical education reforms, career counseling, and mentorship programs. The findings are particularly valuable for addressing the growing global shortage of CCM specialists by offering actionable strategies to attract more medical trainees to this field. The study also emphasizes the importance of integrating early exposure to CCM and strategies for managing lifestyle concerns into medical training, contributing to workforce sustainability and improved healthcare delivery in critical care settings.

## 2 Materials and methods

### 2.1 Study design and population

This cross-sectional study was conducted among medical students at Jazan University, specifically targeting two groups: final-year students and interns. The inclusion criteria were final-year medical students and interns who had completed at least one clinical rotation in CCM. Students who had not undergone CCM rotations or those unwilling to participate were excluded. The study was conducted between January and April 2024.

An *a priori* power analysis was performed using G*Power software to determine the minimum required sample size. The analysis was based on a Chi-squared test, with the following parameters: a medium effect size of w = 0.30, a type I error probability (*α*) of 0.05, a power (1-*β*) of 0.80, and 4 degrees of freedom (df), reflecting the comparison between two educational levels (final-year students and interns) and five categories (five-point Likert scale) of perceptions of critical care specialties. The analysis indicated that a minimum of 133 participants was required to achieve adequate statistical power for detecting medium-sized effects in the Chi-squared test. A total of 165 participants were ultimately included in the study.

Convenience sampling was employed to recruit participants. Invitations were sent via email and WhatsApp messages to groups specifically designated for final-year medical students and interns. The survey was distributed through Google Forms, allowing participants to complete it at their convenience. Follow-up messages were sent to maximize participation and ensure an adequate sample size.

### 2.2 Data collection tool

A self-administered questionnaire was used as the primary data collection instrument, which was divided into demographic information and the factors that had the greatest influence on their career specialty choices and perceptions of the critical care specialty. The survey collected demographic data, including gender, age, marital status, and level of education (final-year student or intern). One specific item asked respondent to select the factors that had the greatest influence on their career specialty choices. Perceptions of the CCM specialty was assessed via 17 items. Responses were recorded on a five-point Likert scale, ranging from 1 (strongly disagree) to 5 (strongly agree). The questionnaire was developed based on a literature review and adapted from a previously validated study (12). The survey was developed based on prior validated instruments from the literature on medical trainees’ perceptions of CCM and was pilot-tested with 10 participants of interns and final-year students to ensure clarity, face validity, and reliability. Based on feedback from the pilot, minor adjustments were made to enhance question clarity. The survey demonstrated good internal consistency, with a Cronbach’s alpha of 0.85.

### 2.3 Ethical considerations

Ethical approval was granted by Jazan University’s ethics committee [Approval No: (HAPO-10-Z^−042^)], and the study adhered to the principles outlined in the Declaration of Helsinki. Informed consent was obtained electronically from all participants before they proceeded with the survey. Participation was voluntary, and confidentiality was ensured by anonymizing all responses.

### 2.4 Data analysis

Data were analyzed using IBM SPSS Statistics (version [26]). For ease of interpretation, responses from the five-point Likert scale were recategorized into three categories: negative perceptions (combining “strongly disagree” and “disagree”), neutral, and positive perceptions (combining “agree” and “strongly agree”). Frequency distributions were used to present the recategorized data. Associations between categorical variables (e.g., gender [male vs. female], educational stage [final-year students vs. interns]) and response distributions were analyzed using Chi-squared tests. Statistical significance was set at *p* < 0.05.

## 3 Results

The demographic composition of the study participants is detailed in Table 1. Of the 165 medical students surveyed, the majority were male (56.36%) and single (90.91%). The average age was 24.56 years (SD = 1.46, range 23 to 29 years). Most participants were interns (80.00%), with a smaller proportion consisting of final-year medical students (20.00%).

**Table 1:** Demographic profile of medical students surveyed.

Item	Variables	Count (%)
Sex	Female	72 (43.64%)
	Male	93 (56.36%)
Age (years)	24.56 ± 1.46 (Range: 23 to 29)	
Marital status	Single	150 (90.91%)
	Married	15 (9.09%)
Current educational stage	Final year	33 (20.00%)
	Internship	132 (80.00%)

Table 2 illustrates the variety of factors influencing medical students’ specialty choices. The most influential factor was the likely availability of career posts, cited by 11.65% of respondents, reflecting a practical approach to job security. Following this, enthusiasm/commitment (what students really want to do) and wanting a career with acceptable hours and working conditions (both at 10.36%) were also key considerations. Future financial prospects (8.41%) and work-life balance factors, such as the ability to raise children (7.77%), were important to many participants. Less influential factors included advice from others (2.27%) and self-appraisal of skills (2.91%).

**Table 2:** Influential factors in medical students’ specialty choice decisions.

Factors influencing your choice of career as a great deal	Count (%)
Likely availability of career posts	108 (11.65%)
Enthusiasm/commitment – what I really want to do	96 (10.36%)
Wanting a career with acceptable hours/working conditions	96 (10.36%)
Future financial prospects	78 (8.41%)
Allows time to raise children	72 (7.77%)
Likely availability of postgraduate training places	57 (6.15%)
Direct patient assistance	54 (5.83%)
Experience of chosen subject as a student	84 (9.06%)
Wanting a career that fits my domestic circumstances	84 (9.06%)
Work daytime only	45 (4.85%)
A particular teacher/department	42 (4.53%)
Career and promotion prospects	33 (3.56%)
Inclinations before medical school	30 (3.24%)
Self-appraisal of own skills/aptitudes	27 (2.91%)
Advice from others	21 (2.27%)

In Table 3, students rated CCM specialties highly for being interesting and challenging (81.82%), advanced (80.00%), and in crisis (78.19%), which suggests a recognition of the field’s importance and urgency. Other favorable perceptions included opportunities for private practice (70.91%) and high salary prospects (76.36%). However, significant concerns were raised about the field’s work-life balance and family time compatibility, with only 32.73% agreeing that the specialty allows for adequate family time. Furthermore, only 29.09% of students were positively considering specializing in CCM.

**Table 3:** Overall perceptions of critical care specialties among participants.

Items	Disagreement	Neutral	Agreement
Interesting and challenging	6 (3.64%)	24 (14.55%)	135 (81.82%)
Advanced specialty	6 (3.64%)	27 (16.36%)	132 (80.00%)
Boring specialty	72 (43.64%)	42 (25.45%)	51 (30.91%)
Stressful specialty	21 (12.73%)	24 (14.55%)	120 (72.73%)
Controllable lifestyle	39 (23.64%)	69 (41.82%)	57 (34.54%)
Possible to work limited hours	39 (23.63%)	63 (38.18%)	63 (38.18%)
Allows for family time	51 (30.91%)	60 (36.36%)	54 (32.73%)
Long working hours	24 (14.55%)	63 (38.18%)	78 (47.28%)
Prestigious (population)	39 (23.64%)	54 (32.73%)	72 (43.64%)
Prestigious (colleagues)	30 (18.19%)	45 (27.27%)	90 (54.54%)
Opportunity for private practice	21 (12.73%)	27 (16.36%)	117 (70.91%)
High salary	9 (5.45%)	30 (18.18%)	126 (76.36%)
Lifestyle vs. income	27 (16.36%)	63 (38.18%)	75 (45.46%)
Academic opportunities	9 (5.45%)	57 (34.55%)	99 (60.00%)
Other students consider	48 (29.09%)	51 (30.91%)	66 (40.00%)
Specialty in crisis	12 (7.27%)	24 (14.55%)	129 (78.19%)
Specialty I am positively considering	72 (43.64%)	45 (27.27%)	48 (29.09%)

Table 4 presents significant differences in perceptions based on educational level. Interns were more likely to view the availability of limited working hours positively (*p* = 0.046) compared to final-year students. Additionally, interns perceived greater prestige among colleagues (*p* = 0.035) and better opportunities for private practice (*p* = 0.004). They also expected higher salaries (*p* < 0.001) and acknowledged the shortage in staff (*p* = 0.009). These findings suggest that internship experiences may foster a more favorable outlook on career opportunities in CCM. There were no statistically significant differences in perceptions between male and female participants across the measured variables.

**Table 4:** Gender and educational level differences in perceptions of critical care specialty among participants.

Items	Female (*N* = 72)	Male (*N* = 93)	*p*-value	Internship (*N* = 132)	Final Year (*N* = 33)	*p*- value
Interesting and challenging	79.1% (57)	83.8% (78)	0.104	84.1% (111)	72.7% (24)	0.538
Advanced specialty	79.1% (57)	80.6% (75)	0.695	81.9% (108)	72.7% (24)	0.349
Boring specialty	29.1% (21)	32.3% (30)	0.532	31.9% (42)	27.3% (9)	0.899
Stressful specialty	70.9% (51)	74.2% (69)	0.984	72.7% (96)	72.7% (24)	0.761
Controllable lifestyle	29.2% (21)	38.8% (36)	0.544	31.8% (42)	45.5% (15)	0.586
Possible to work limited hours	29.1% (21)	45.2% (42)	0.408	36.4% (48)	45.5% (15)	**0.046**
Allows for family time	29.2% (21)	35.5% (33)	0.685	29.6% (39)	45.5% (15)	0.626
Long working hours	45.8% (33)	48.4% (45)	0.699	45.5% (60)	54.6% (18)	0.203
Prestigious (public)	37.5% (27)	48.4% (45)	0.796	52.3% (69)	9.10% (3)	0.124
Prestigious (colleagues)	41.7% (30)	64.5% (60)	0.123	63.7% (84)	18.2% (6)	**0.035**
Opportunity for private practice	58.3% (42)	80.6% (75)	0.165	79.5% (105)	36.4% (12)	**0.004**
High salary	79.1% (57)	74.2% (69)	0.070	79.6% (105)	63.6% (21)	**0.001**
Lifestyle vs. income	50.0% (36)	41.9% (39)	0.160	47.7% (63)	36.4% (12)	0.179
Academic opportunities	66.7% (48)	54.8% (51)	0.553	63.7% (84)	45.5% (15)	0.107
Other students consider	33.3% (24)	45.2% (42)	0.511	43.2% (57)	27.3% (9)	0.754
Specialty in crisis	75.0% (54)	80.6% (75)	0.263	84.1% (111)	27.3% (9)	**0.009**
Specialty I am positively considering	37.5% (27)	22.6% (21)	0.812	27.3% (36)	36.4% (12)	0.970

Values are the percentage of agree/strongly agree answers on a 5 point-Likert scale.

## 4 Discussion

The findings from this study provide critical insights into the factors influencing medical students’ specialty choices, their perceptions of CCM specialties, and how these views differ across educational levels. Understanding these factors is essential for addressing workforce distribution, specialty shortages, and ensuring a well-prepared future medical workforce.

This study provides important insights into the factors medical students prioritize when choosing a specialty. Job security (11.65%) emerged as the most influential factor when students considered their specialty choice, reflecting a pragmatic approach to career stability. Enthusiasm/commitment (10.36%) and the desire for acceptable working hours and conditions (10.36%) were equally important, highlighting the value students place on personal satisfaction and work-life balance. These findings align with global trends, where lifestyle factors increasingly dominate specialty decision-making (13, 14). Interestingly, financial incentives (8.41%) and family planning (7.77%) were less influential, indicating that while students care about future earnings, they may prioritize sustainable work conditions and personal well-being over purely financial considerations. This aligns with studies showing that lifestyle factors tend to outweigh financial incentives and external advice when selecting a specialty (15).

Perceptions of CCM revealed a complex view. A significant majority of students rated CCM as intellectually challenging (81.82%) and advanced (80.00%), with 78.19% recognizing its role in addressing urgent healthcare needs. These perceptions are consistent with prior research, where CCM is seen as stimulating and central to modern healthcare (12). However, the demanding nature of the field generated concerns about work-life balance. Only 32.73% of students felt CCM allows for adequate family time, and just 34.54% believed it offers a controllable lifestyle. These concerns mirror findings by Mehmood et al. and reflect the inherent tension in specialties requiring long working hours and emotional resilience (16). This tension likely explains why only 29.09% of students expressed serious interest in pursuing CCM. Despite its intellectual appeal and potential financial rewards, the perception of an uncontrollable lifestyle deters students from choosing this specialty. This pattern is consistent with other research indicating that lifestyle considerations are key deterrents even in highly rewarding specialties (12).

There is a clear distinction between the factors influencing overall specialty choices and the specific perceptions of CCM. While general specialty choices are primarily driven by pragmatic concerns like job security (11.65%) and work-life balance (10.36%), students’ perceptions of CCM are shaped more by its intellectual demands and its essential role in healthcare. For example, CCM is widely recognized as intellectually challenging (81.82%) and advanced (80.00%), reflecting its appeal to those seeking complex, high-stakes environments. However, despite the intellectual appeal of CCM, students’ general priorities—such as a desire for manageable working hours and career sustainability—are less aligned with the demanding nature of the specialty. This contrast helps explain the relatively low interest in CCM despite its perceived importance, as students value lifestyle factors and job stability over the rigorous and emotionally taxing demands of CCM.

Significant differences in perceptions were observed between interns and final-year students, reflecting how educational progression influences career outlooks. Compared to students, interns viewed limited working hours more favorably (*p* = 0.046), perceived greater private practice opportunities (*p* = 0.004), and anticipated higher salaries (*p* < 0.001). Additionally, interns were more likely to acknowledge the shortage of staff in CCM (*p* = 0.009), which may reflect their exposure to the demands and resource limitations in clinical settings. The overall optimism of interns is likely driven by their proximity to transitioning into full-time practice, where financial opportunities become more tangible (17). However, despite these positive views, interns still expressed reservations about CCM as a specialty, particularly regarding staffing challenges. This aligns with findings from a previous study, which noted that interns tend to have more optimistic expectations about their careers than final-year students, likely due to their evolving understanding of specialty demands as they gain hands-on experience (18). Thus, while internship experiences may foster a more favorable outlook on financial and career opportunities, they also provide a clearer perspective on the realities of working in resource-constrained environments like CCM. Interestingly, the study found no statistically significant differences between male and female participants in their perceptions of CCM specialties. This contrasts with prior research that has reported gender differences in specialty preferences, particularly regarding lifestyle factors (12, 19).

### 4.1 Implications for medical education and career guidance

The findings have important implications for medical education and career counseling. The divergence in perceptions between students and interns highlights the need for realistic career guidance early in medical training. Career counseling programs should address the lifestyle concerns associated with high-demand specialties like CCM and provide a balanced view of the professional rewards and personal sacrifices involved. Medical educators could also integrate early mentorship programs that expose students to the realities of CCM earlier in their education. This would help mitigate misconceptions contributing to low interest in the field. Additionally, offering work-life balance strategies and discussing emotional resilience within medical curricula may better prepare students for the demands of fields like CCM. By fostering a more nuanced understanding of both the rewards and challenges of CCM, educators could increase student interest in this high-demand specialty.

### 4.2 Limitations and future directions

This study has several limitations. The use of a convenience sampling method limits the generalizability of the findings, as the sample may not represent all medical students and interns in Saudi Arabia. Additionally, the self-reported nature of the survey data could introduce social desirability bias, where participants may respond in a way they believe is expected rather than providing their true opinions. Future research should aim to use probability sampling methods to improve generalizability and explore the longitudinal effects of clinical exposure on students’ perceptions and career choices. Furthermore, examining how socioeconomic background, personal motivations, and regional healthcare systems influence specialty choices could provide a deeper understanding of the factors shaping medical students’ career trajectories.

## 5 Conclusion

This study provides key insights into the factors influencing medical students’ specialty choices, with job security, enthusiasm/commitment, and work-life balance emerging as top priorities. While CCM was valued for its intellectual challenges and financial rewards, concerns about work-life balance and lifestyle controllability limited students’ interest in pursuing the specialty. The differences between interns and final-year students suggest that increased clinical exposure leads to more optimistic views of career opportunities but does not alleviate concerns about the demands of CCM. Addressing these lifestyle concerns through early mentorship and targeted career counseling could help attract more students to high-demand specialties like CCM. Future research should explore the long-term effects of clinical exposure and examine the role of cultural and regional factors in shaping specialty preferences.

## Data Availability

The raw data supporting the conclusions of this article will be made available by the authors, without undue reservation.
